# Association Between Socioeconomic Status and Incident Sarcopenic Obesity: A 17-Year Prospective Cohort Study

**DOI:** 10.3390/jcm15103816

**Published:** 2026-05-15

**Authors:** Hye Rang Bak, Nak Gyeong Ko, Hyun-Min Koh, Ji-Yong Jang, Jeong Gyu Lee, Yu Hyeon Yi, Seunghun Lee, Duk-Young Cho, Young Hye Cho

**Affiliations:** 1 Department of Family Medicine, Samsung Changwon Hospital, Sungkyunkwan University School of Medicine, Changwon 51353, Republic of Korea; hyerangbak@gmail.com (H.R.B.); sunkhm@naver.com (H.-M.K.); aeo-lus7@naver.com (J.-Y.J.); 2 Department of Family Medicine, Pusan National University School of Medicine, Yangsan 50612, Republic of Korea; jeklee@pusan.ac.kr (J.G.L.); eeugus@hanmail.net (Y.H.Y.); greatseunghun@hanmail.net (S.L.); 3 Department of Research & Support, Samsung Changwon Hospital, Sungkyunkwan University School of Medicine, Changwon 51353, Republic of Korea; nakgyeong.ko@samsung.com; 4 Department of Family Medicine and Biomedical Research Institute, Pusan National University Hospital, Busan 49241, Republic of Korea; 5 Department of Medical Management, Pusan National University School of Medicine, Yangsan 50612, Republic of Korea; dycho@pusan.ac.kr; 6 Department of Family Medicine and Biomedical Research Institute, Pusan National University Yangsan Hospital, Yangsan 50612, Republic of Korea

**Keywords:** sarcopenic obesity, socioeconomic status, longitudinal study

## Abstract

**Background:** Sarcopenic obesity, defined as the coexistence of low muscle mass and excess adiposity, is an emerging public health concern in aging populations. However, longitudinal evidence on the association between socioeconomic status (SES) and incident sarcopenic obesity remains limited, particularly in Asian populations. This study aimed to investigate the association between SES and the long-term incidence of sarcopenic obesity in a large Korean cohort. **Methods:** We conducted a prospective cohort study using data from the Korean Genome and Epidemiology Study (KoGES). Community-dwelling adults aged ≥ 40 years without sarcopenic obesity at baseline were followed for a mean of 17 years. Sarcopenic obesity was defined as low body mass index (BMI)-adjusted skeletal muscle mass (sex-specific lowest 20th percentile) combined with obesity (BMI ≥ 25 kg/m^2^). SES indicators included educational attainment, household income, and marital status. Kaplan–Meier methods were used to estimate cumulative incidence, and Cox proportional hazards models were applied to calculate hazard ratios (HRs) and 95% confidence intervals (CIs) with sequential adjustment for demographic, lifestyle, and metabolic factors. **Results:** In the fully adjusted model, participants with elementary school education showed a higher hazard ratio of incident sarcopenic obesity compared with those with college education or higher (HR 1.39; 95% CI 1.11–1.73; *p* = 0.003). Similarly, individuals with a household income < 1.0 million KRW per month had a higher hazard ratio compared with those earning ≥4.0 million KRW (HR 1.31; 95% CI 1.02–1.70; *p* = 0.037). Unmarried participants also showed a higher hazard ratio compared with married individuals (HR 1.31; 95% CI 1.09–1.57; *p* = 0.003). **Conclusions:** Lower SES was independently associated with a higher incidence of sarcopenic obesity over long-term follow-up in this population-based cohort. These findings highlight the importance of considering socioeconomic factors in the prevention of sarcopenic obesity.

## 1. Introduction

Sarcopenia, an age-related condition characterized by progressive declines in skeletal muscle mass, strength, and physical performance, represents an important contributor to functional impairment and reduced quality of life among older adults [1,2]. With rapid global population aging, the prevalence of sarcopenia has increased substantially, reaching up to 29% among community-dwelling older adults in some studies [3]. At the same time, the prevalence of overweight and obesity has risen worldwide. According to the World Obesity Atlas 2023, the global prevalence of overweight and obesity was estimated at 38% in 2020 and is projected to reach 51% by 2035 [4]. These concurrent trends are expected to further increase the prevalence of sarcopenic obesity, a condition characterized by the coexistence of low muscle mass and excess adiposity [5].

Sarcopenic obesity represents more than a simple alteration in body composition; it is associated with a wide range of adverse health outcomes, including functional decline, increased risk of falls and fractures, cardiometabolic disease, and increased mortality [6,7]. As global populations age and the prevalence of obesity continues to rise, the burden of sarcopenic obesity is expected to increase. These trends highlight the growing clinical and public health importance of identifying modifiable determinants of sarcopenic obesity. The development of sarcopenic obesity is influenced by a complex interplay of biological, behavioral, and environmental factors [8]. Older age, sex, and chronic comorbid conditions are well-established determinants of body composition changes [9,10]. In parallel, lifestyle factors such as physical inactivity, smoking, excessive alcohol consumption, and inadequate dietary protein intake have also been identified as important contributors [11,12]. However, these individual-level factors are not randomly distributed across populations but are patterned by broader social and economic conditions.

Socioeconomic status (SES), a multidimensional construct encompassing education, income, and marital status, is a key determinant of health inequalities across populations [13]. Individuals in socioeconomically disadvantaged conditions often face barriers to maintaining healthy diets, engaging in regular physical activity, and accessing preventive healthcare services [14,15]. In addition to these behavioral pathways, socioeconomic disadvantage may also affect biological processes, including stress-related and metabolic dysregulation, thereby contributing to the development of sarcopenic obesity [16].

Previous studies have extensively examined the associations of socioeconomic status with sarcopenia and obesity separately across diverse populations [17,18]. In contrast, research on sarcopenic obesity is limited and largely cross-sectional in design, restricting causal inference [19,20]. Consequently, longitudinal evidence on this relationship remains scarce, particularly in Asian populations. To address this gap, the present study used data from the Korean Genome and Epidemiology Study (KoGES), a large community-based prospective cohort, to evaluate the long-term association between key socioeconomic indicators—educational attainment, household income, and marital status—and incident sarcopenic obesity over a mean follow-up of 17 years. We hypothesized that lower socioeconomic status would be associated with a higher incidence of sarcopenic obesity, independent of demographic characteristics, lifestyle factors, and metabolic comorbidities.

## 2. Materials and Methods

### 2.1. Study Design and Data Source

This prospective longitudinal study used data from the KoGES, a large government-supported population-based cohort conducted by the Korea Disease Control and Prevention Agency (KDCA) and the National Institute of Health (NIH). The community-based Ansan and Anseong cohorts enrolled adults aged ≥40 years between 2001 and 2002 and conducted follow-up examinations biennially through 2018, resulting in a total of eight follow-up waves. Participants were recruited voluntarily through telephone contact, mailed invitations, community registration, and media campaigns, and underwent standardized physical examinations and structured questionnaires at designated university hospitals or certified health examination centers. Detailed descriptions of the study design and survey procedures have been published previously [21].

Among participants who were free of sarcopenic obesity at baseline (*n* = 8797), individuals who participated in only a single follow-up examination (*n* = 773) were excluded to ensure adequate follow-up, leaving 8024 participants for the final analysis. All study procedures were conducted in accordance with the ethical principles of the Declaration of Helsinki, and all participants provided written informed consent. The present study was approved by the Institutional Review Board of Samsung Changwon Hospital (IRB No. SCMC IRB 2024-12-015-001).

### 2.2. Assessment of Socioeconomic Characteristics and Health Behaviors

Baseline socioeconomic characteristics and lifestyle behaviors were collected using standardized self-administered questionnaires provided by KoGES, and baseline values were used to define exposure variables.

SES was assessed using educational attainment, household income, and marital status. Educational attainment was categorized as elementary school or less, middle-high school, and college or higher. Monthly household income was classified into three groups: <1.0 million Korean won (KRW), 1.0–3.9 million KRW, and ≥4.0 million KRW. Marital status was categorized as married or unmarried, with the unmarried group including single, divorced, separated, and widowed individuals.

Lifestyle variables included smoking status, alcohol consumption, physical activity, and dietary intake. Smoking status was categorized as non-smoker, ex-smoker, or current smoker. Alcohol consumption was classified as non-drinker, ex-drinker, or current drinker. Physical activity was defined based on participation in moderate-intensity physical activity for at least 30 min per day; detailed resistance exercise information was not used due to low response rates. Dietary intake was assessed using a semi-quantitative food frequency questionnaire, from which average daily total energy (kcal), protein (g), fat (g), and carbohydrate (g) intakes were calculated.

### 2.3. Anthropometric and Clinical Measurements

Anthropometric measurements were performed by trained medical personnel using standardized protocols. Body weight was measured to the nearest 0.1 kg, and height was measured without shoes. Body mass index (BMI) was calculated as weight (kg) divided by height squared (m^2^). Waist circumference was measured at the midpoint between the lowest rib and the iliac crest, and hip circumference was measured at the widest portion of the buttocks to calculate the waist-to-hip ratio.

Blood pressure was measured in the seated position after at least 5 min of rest. Measurements were taken from both arms, and the average value of repeated measurements (at least twice at 30 s intervals) from the arm with the higher reading was used for analysis.

Blood samples were collected after at least 8 h of overnight fasting, and fasting glucose, glycated hemoglobin (HbA1c), total cholesterol, high-density lipoprotein cholesterol (HDL-C), low-density lipoprotein cholesterol (LDL-C), and triglycerides were measured using an automated analyzer (ADVIA 1650; Siemens, Tarrytown, NY, USA).

Chronic diseases were defined as follows. Hypertension was defined as systolic blood pressure ≥ 140 mmHg, diastolic blood pressure ≥ 90 mmHg, or self-reported physician diagnosis. Diabetes mellitus was defined as fasting glucose ≥ 126 mg/dL, HbA1c ≥ 6.5%, or physician diagnosis. Dyslipidemia was defined as LDL cholesterol ≥ 130 mg/dL, HDL cholesterol < 40 mg/dL, or use of lipid-lowering medications.

### 2.4. Definition of Sarcopenia, Obesity, and Sarcopenic Obesity

Because direct measurements of muscle strength or physical performance were not available in KoGES, sarcopenia was operationally defined using sex-specific relative skeletal muscle mass. Body composition, including skeletal muscle mass, fat mass, and lean mass, was measured using a multi-frequency bioelectrical impedance analyzer (MF-BIA; InBody 3.0, Biospace, Seoul, Republic of Korea).

The skeletal muscle mass index was calculated as skeletal muscle mass (kg) divided by BMI (kg/m^2^) (SMM/BMI). Participants with values below the sex-specific 20th percentile were classified as having low muscle mass. This approach is consistent with previous studies, including the Foundation for the National Institutes of Health (FNIH) Sarcopenia Project, which proposed body mass index-adjusted skeletal muscle mass indices as a clinically relevant measure of sarcopenia [22]. In addition, BMI-adjusted skeletal muscle indices have been widely applied in population-based studies, including those conducted in Asian populations [23,24].

Obesity was defined as BMI ≥ 25 kg/m^2^ according to the Asia-Pacific World Health Organization criteria [25]. Participants who met both the low muscle mass and obesity criteria were classified as having sarcopenic obesity.

This operational definition was selected to ensure applicability within the available dataset and comparability with previous population-based studies.

### 2.5. Statistical Analysis

Baseline characteristics of study participants were summarized as means ± standard deviations for continuous variables and frequencies (percentages) for categorical variables. Differences between groups according to socioeconomic status were compared using independent *t*-tests or analysis of variance (ANOVA) for continuous variables and chi-square tests for categorical variables.

The cumulative incidence of sarcopenic obesity was estimated using the Kaplan–Meier method, and differences between socioeconomic groups were assessed using the log-rank test. Multivariable Cox proportional hazards regression models were used to estimate hazard ratios (HRs) and 95% confidence intervals (CIs) for incident sarcopenic obesity.

Sequential adjustment models were constructed as follows: Model 1 was adjusted for age and sex; Model 2 was additionally adjusted for lifestyle factors (smoking, alcohol consumption, physical activity, and protein intake); and Model 3 further adjusted for metabolic comorbidities (hypertension, dyslipidemia, and diabetes mellitus) to evaluate the independent association between socioeconomic status and incident sarcopenic obesity.

Missing data were handled using a complete-case analysis approach. Given the relatively low proportion of missing values, this approach was considered unlikely to introduce substantial bias.

All statistical analyses were performed using R statistical software (version 4.3.0; R Foundation for Statistical Computing, Vienna, Austria), and statistical significance was defined as a two-sided *p*-value < 0.05.

## 3. Results

### 3.1. Baseline Characteristics of Study Participants

A total of 8024 adults who were free of sarcopenic obesity at baseline were included in the final analysis (Table 1). The mean age of participants was 51.8 ± 8.8 years, and 3854 (48.0%) were men and 4170 (52.0%) were women.

Regarding socioeconomic characteristics, participants with middle or high school education constituted the largest proportion (54.6%), whereas 13.8% had a college education or higher. For household income, the majority of participants (57.9%) were in the 1.0–3.9 million KRW group. Most participants were married (91.0%).

Baseline characteristics according to socioeconomic status are presented in Supplementary Tables S1–S3. In addition, subgroup analyses stratified by sex and age are presented in Supplementary Tables S4–S6.

### 3.2. Cumulative Incidence of Sarcopenic Obesity According to Socioeconomic Status

**Figure 1:**
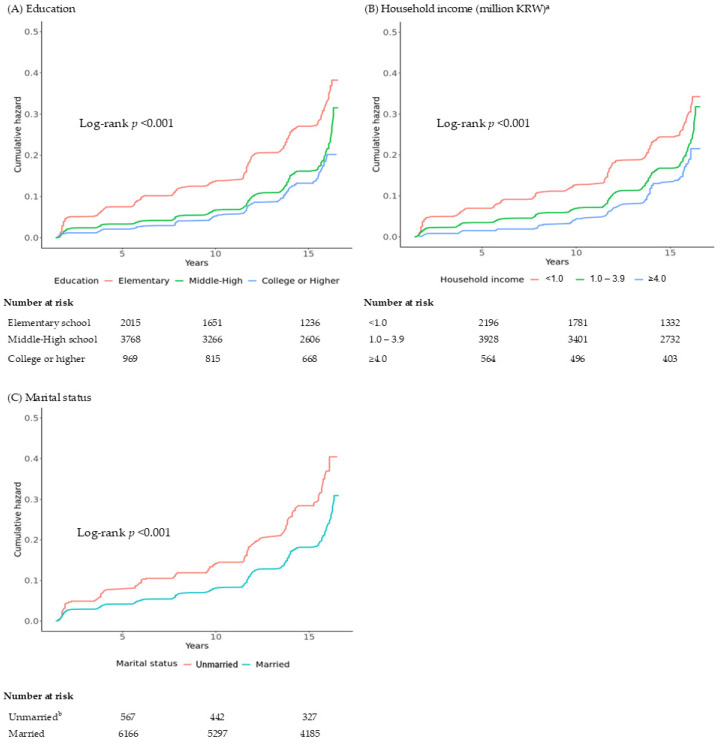
Kaplan–Meier curves showing the cumulative incidence of sarcopenic obesity according to (**A**) education level, (**B**) household income, and (**C**) marital status among 8024 Korean adults during a mean follow-up of 17 years. ^a^ For example, 4.0 = 4,000,000 KRW. ^b^ Unmarried status included individuals who were single, divorced, separated, or widowed. Abbreviation: KRW, Korean won.

Table 2 and Figure 1 present the cumulative incidence and hazard ratios for incident sarcopenic obesity according to socioeconomic status.

The cumulative incidence was the highest in the lowest educational group (21.2%) and the lowest in the highest educational group (12.0%). Compared with the highest education group, the lowest education group showed a significantly increased risk (HR 1.93; 95% CI 1.60–2.34; *p* < 0.001), whereas the middle education group was not significantly associated (HR 1.16; 95% CI 0.96–1.39). A similar gradient was observed across household income levels, with the highest cumulative incidence in the lowest-income group (19.3%) and the lowest incidence in the highest-income group (12.5%). The lowest-income group had a significantly elevated risk compared with the highest-income group (HR 1.83; 95% CI 1.44–2.33; *p* < 0.001). Unmarried individuals demonstrated a higher cumulative incidence (21.7%) than married individuals (15.6%), corresponding to a significantly increased risk (HR 1.62; 95% CI 1.37–1.91; *p* < 0.001). These patterns were visually consistent in the Kaplan–Meier curves, which showed progressively steeper cumulative incidence trajectories with decreasing socioeconomic status.

### 3.3. Multivariable-Adjusted Risk of Sarcopenic Obesity

Table 3 presents the associations between socioeconomic status and incident sarcopenic obesity across sequentially adjusted models. Model 1 was adjusted for age and sex, Model 2 additionally adjusted for lifestyle factors, and Model 3 further adjusted for comorbidities.

Lower educational attainment remained significantly associated with higher risk after full adjustment. Compared with participants with college education or higher, those with elementary education or less had an increased risk (HR 1.39, 95% CI: 1.11–1.73; *p* = 0.003), whereas the association for the middle education group was not statistically significant. Lower household income also remained independently associated with increased risk in the fully adjusted model (HR 1.31, 95% CI: 1.02–1.70; *p* = 0.037 for the lowest income group). Similarly, unmarried individuals showed a significantly higher risk compared with married individuals (HR 1.31, 95% CI: 1.09–1.57; *p* = 0.003). These findings indicate that the association between socioeconomic disadvantage and sarcopenic obesity persisted even after adjustment for potential confounders.

## 4. Discussion

In this large community-based prospective cohort study of Korean adults, lower socioeconomic status was associated with a higher incidence of sarcopenic obesity over a mean follow-up of 17 years. These associations remained after adjustment for demographic characteristics, lifestyle factors, and metabolic comorbidities, and were in line with findings from previous longitudinal studies. Unlike many previous studies that relied on cross-sectional designs or examined sarcopenia or obesity separately [9,26], this study evaluated the combined phenotype of sarcopenic obesity using a long-term longitudinal framework, providing further evidence on the temporal relationship between socioeconomic disadvantage and adverse body composition outcomes. Furthermore, the use of a large Asian population-based cohort adds important epidemiological evidence from an understudied population.

Different components of socioeconomic status may reflect distinct dimensions of health inequality. Although individuals in higher socioeconomic groups tended to be younger and more likely to be male (Supplementary Tables S1–S3), the associations between socioeconomic disadvantage and sarcopenic obesity remained largely unchanged after sequential adjustment for demographic factors, lifestyle behaviors, and comorbidities. These findings suggest that age and sex differences alone do not fully explain the observed associations.

Several explanations may account for the association between low socioeconomic status and sarcopenic obesity. Educational attainment may be related to health literacy and the ability to adopt and maintain healthy behaviors [27]. Household income may influence access to nutritional resources, opportunities for physical activity, and healthcare utilization [28]. Marital status, as a proxy for social support, may also be associated with long-term health behaviors through shared lifestyle patterns, emotional support, and encouragement for healthcare utilization and healthy daily routines [29]. Taken together, these findings suggest that multiple dimensions of socioeconomic disadvantage may be linked to the development of sarcopenic obesity. However, these findings should be interpreted with caution. This is because reverse causation cannot be excluded, as individuals with poorer baseline health may be more likely to experience socioeconomic disadvantage. In addition, residual confounding from unmeasured factors, such as mental health [30,31], dietary quality [32], or genetic predisposition [33], may have influenced the observed associations.

The definition of sarcopenia varies across consensus groups, including those proposed by the European Society for Clinical Nutrition and Metabolism (ESPEN) and European Association for the Study of Obesity (EASO) and the Asia–Oceania consensus, and no single standardized criterion has been universally adopted [8,34]. Notably, in the recent Global Leadership Initiative in Sarcopenia (GLIS) consensus, physical performance is no longer considered a core component of sarcopenia but rather an outcome of the condition [35]. In large-scale epidemiological studies, relative and population-specific cutoffs are often applied instead of universal thresholds, particularly when ethnic differences exist or when direct measurements of muscle strength or physical performance are unavailable [36,37]. In this study, sex-specific lower 20th percentile values of BMI-adjusted skeletal muscle mass were used to define low muscle mass to ensure internal comparability and reproducibility within the cohort. Therefore, the present definition should be interpreted as a population-based relative sarcopenic obesity phenotype rather than a clinically diagnosed sarcopenic obesity according to current consensus guidelines.

The strengths of this study include the use of a large, community-based prospective cohort with an extended follow-up period of approximately 17 years, the multidimensional assessment of socioeconomic status, and comprehensive adjustment for lifestyle factors and major metabolic comorbidities. However, several limitations should be acknowledged. First, socioeconomic status variables were assessed only at baseline and may not capture changes over the follow-up period, potentially leading to exposure misclassification. Second, body composition was measured using bioelectrical impedance analysis rather than imaging-based techniques, which may be subject to measurement error. Third, direct measures of muscle strength and physical performance were not available, limiting the application of consensus-based diagnostic criteria for sarcopenia.

Future studies incorporating direct measures of muscle strength or physical performance are warranted to further refine the definition and clinical relevance of sarcopenic obesity. In addition, repeated socioeconomic assessments and time-dependent longitudinal analyses may improve understanding of the dynamic relationships between socioeconomic disadvantage and adverse body composition changes over time. Further research is also needed to clarify the underlying mechanisms and potential causal pathways linking socioeconomic status and sarcopenic obesity.

## 5. Conclusions

In conclusion, lower socioeconomic status was independently associated with a higher incidence of sarcopenic obesity in this large population-based cohort of Korean adults. These findings suggest that sarcopenic obesity may be considered not solely as a consequence of biological aging but also as a manifestation of accumulated socioeconomic disadvantage across the life course. Public health strategies aimed at preventing sarcopenic obesity should therefore extend beyond individual-level lifestyle interventions to include population-level approaches that address socioeconomic vulnerability.

## Figures and Tables

**Table 1:** Baseline characteristics of study participants (*n* = 8024).

Characteristics	
Age (y)	51.8 ± 8.8
Sex	
Male	3854 (48.0)
Female	4170 (52.0)
Height (cm)	160.8 ± 8.5
Weight (kg)	62.5 ± 10.2
BMI (kg/m^2^)	24.1 ± 2.9
Waist circumference (cm)	81.7 ± 8.5
Waist/Hip ratio	0.9 ± 0.0
Body fat mass (kg)	16.1 ± 5.1
Body fat mass (%)	25.8 ± 6.9
Skeletal muscle mass (kg)	43.7 ± 8.2
SMM/BMI	1.8 ± 0.3
Lean body mass (kg)	46.3 ± 8.6
Systolic BP (mmHg)	120.6 ± 18.1
Diastolic BP (mmHg)	79.8 ± 11.3
Glucose (mg/dL)	86.6 ± 20.4
HbA1c (%)	5.8 ± 0.9
Total cholesterol (mg/dL)	189.7 ± 35.2
HDL cholesterol (mg/dL)	44.9 ± 10.1
LDL cholesterol (mg/dL)	113.5 ± 32.3
Triglyceride (mg/dL)	158.5 ± 110.9
Hypertension	2383 (29.7)
Diabetes mellitus	894 (11.2)
Dyslipidemia	186 (2.3)
Drinking status	
Non-drinker	3617 (45.5)
Ex-drinker	492 (6.2)
Current drinker	3846 (48.4)
Smoking status	
Non-smoker	4597 (58.0)
Ex-smoker	1234 (15.6)
Current smoker	2095 (26.4)
Exercise	
Yes	2911 (37.4)
No	4879 (62.6)
Caloric intake (kcal)	1964.9 ± 717.6
Protein intake (g)	66.9 ± 30.8
Fat intake (g)	32.9 ± 21.8
Carbohydrate intake (g)	345.4 ± 119.5
Education	
Elementary school	2515 (31.6)
Middle-High school	4344 (54.6)
College or higher	1101 (13.8)
Household income (million KRW) ^a^	
<1.0	2702 (34.3)
1.0–3.9	4570 (57.9)
≥4.0	617 (7.8)
Marital status	
Unmarried ^b^	716 (9.0)
Married	7247 (91.0)

Abbreviations: BMI, body mass index; SMM/BMI, BMI-adjusted skeletal muscle mass index; BP, blood pressure; HbA1c, glycated hemoglobin; KRW, Korean won. Chronic diseases were defined as follows: hypertension (systolic blood pressure ≥ 140 mmHg, diastolic blood pressure ≥ 90 mmHg, or physician diagnosis); diabetes mellitus (fasting plasma glucose ≥ 126 mg/dL, HbA1c ≥ 6.5%, or physician diagnosis); dyslipidemia (LDL cholesterol ≥ 130 mg/dL, HDL cholesterol < 40 mg/dL, or use of lipid-lowering medications). Data are presented as mean ± standard deviation for continuous variables and number (percentage) for categorical variables. ^a^ For example, 4.0 = 4,000,000 KRW. ^b^ Unmarried status included individuals who were single, divorced, separated, or widowed.

**Table 2:** Hazard ratios for new onset of sarcopenic obesity by socioeconomic status ^a^.

	Event	HR (95% CI)	*p*-Value
Education			
Elementary school	534 (21.2)	1.93 (1.60, 2.34)	<0.001
Middle-High school	615 (14.2)	1.16 (0.96, 1.39)	0.133
College or higher	132 (12.0)	reference	
Household income (million KRW) ^b^			
<1.0	522 (19.3)	1.83 (1.44, 2.33)	<0.001
1.0–3.9	672 (14.7)	1.26 (0.99, 1.59)	0.059
≥4.0	77 (12.5)	reference	
Marital status			
Unmarried ^c^	155 (21.7)	1.62 (1.37, 1.91)	<0.001
Married	1130 (15.6)	reference	

Abbreviation: HR, hazard ratio; CI, confidence interval; KRW, Korean won. Data are presented as number (percentage) and hazard ratio with 95% confidence interval. ^a^ Event indicates the number of cases. ^b^ For example, 4.0 = 4,000,000 KRW. ^c^ Unmarried status included individuals who were single, divorced, separated, or widowed. *p*-values were derived from the Wald tests in Cox proportional hazards models.

**Table 3:** Multivariable-adjusted hazard ratios for new onset of sarcopenic obesity by socioeconomic status ^a^.

	Event	Model 1		Model 2		Model 3	
		HR (95% CI)	*p*-Value	HR (95% CI)	*p*-Value	HR (95% CI)	*p*-Value
Education							
Elementary school	534 (21.2)	1.45 (1.17, 1.79)	<0.001	1.42 (1.14, 1.76)	0.002	1.39 (1.11, 1.73)	0.003
Middle-High school	615 (14.2)	1.06 (0.88, 1.29)	0.533	1.07 (0.88, 1.30)	0.505	1.07 (0.88, 1.31)	0.478
College or higher	132 (12.0)	reference		reference		reference	
Household income (million KRW) ^b^							
<1.0	522 (19.3)	1.36 (1.06, 1.75)	0.016	1.34 (1.04, 1.73)	0.025	1.31 (1.02, 1.70)	0.037
1.0–3.9	672 (14.7)	1.15 (0.91, 1.46)	0.241	1.12 (0.88, 1.42)	0.355	1.12 (0.88, 1.43)	0.349
≥4.0	77 (12.5)	reference		reference		reference	
Marital status							
Unmarried ^c^	155 (21.7)	1.31 (1.10, 1.56)	0.002	1.31 (1.09, 1.57)	0.003	1.31 (1.09, 1.57)	0.003
Married	1130 (15.6)	reference		reference		reference	

Abbreviation: HR, hazard ratio; CI, confidence interval; KRW, Korean won. Data are presented as number (percentage) and hazard ratio with 95% confidence interval. ^a^ Event indicates the number of cases. ^b^ For example, 4.0 = 4,000,000 KRW. ^c^ Unmarried status included individuals who were single, divorced, separated, or widowed. *p*-values were derived from the Wald test in Cox proportional hazards models. Model 1: adjusted for age, sex. Model 2: adjusted for age, sex, smoking, drinking, exercise and daily protein intake. Model 3: adjusted for age, sex, smoking, drinking, exercise, daily protein intake, hypertension, dyslipidemia and diabetes mellitus.

## Data Availability

The data presented in this study are available within the article and Supplementary Materials.

## Supplementary Materials

The following supporting information can be downloaded at: https://www.mdpi.com/article/10.3390/jcm15103816/s1, Supplementary Table S1. Baseline characteristics according to educational attainment; Supplementary Table S2. Baseline characteristics according to household income categories (million KRW); Supplementary Table S3. Baseline characteristics according to marital status; Supplementary Table S4. Multivariable-adjusted hazard ratios for new onset of sarcopenic obesity by socioeconomic status in men; Supplementary Table S5. Multivariable-adjusted hazard ratios for new onset of sarcopenic obesity by socioeconomic status in women; Supplementary Table S6. Multivariable-adjusted hazard ratios for new onset of sarcopenic obesity by socioeconomic status by age group.

## Abbreviations

The following abbreviations are used in this manuscript:

ANOVA	Analysis of variance
AWGS	Asian Working Group for Sarcopenia
BMI	Body mass index
BP	Blood pressure
CI	Confidence interval
EWGSOP	European Working Group on Sarcopenia in Older People
FNIH	Foundation for the National Institutes of Health
GLIS	Global Leadership Initiative on Sarcopenia
HbA1c	Glycated hemoglobin
HDL	High-density lipoprotein
HR	Hazard ratio
KDCA	Korea Disease Control and Prevention Agency
KoGES	Korean Genome and Epidemiology Study
KRW	Korean won
LDL	Low-density lipoprotein
MF-BIA	Multi-frequency bioelectrical impedance analyzer
NIH	National Institute of Health
SES	Socioeconomic status
SMM	Skeletal muscle mass
SMM/BMI	BMI-adjusted skeletal muscle mass index

## References

[1] Chen L.-K., Woo J., Assantachai P., Auyeung T.-W., Chou M.-Y., Iijima K., Jang H.C., Kang L., Kim M., Kim S. (2020). Asian Working Group for Sarcopenia: 2019 Consensus Update on Sarcopenia Diagnosis and Treatment. J. Am. Med. Dir. Assoc..

[2] Cruz-Jentoft A.J., Bahat G., Bauer J., Boirie Y., Bruyère O., Cederholm T., Cooper C., Landi F., Rolland Y., Sayer A.A. (2019). Sarcopenia: Revised European consensus on definition and diagnosis. Age Ageing.

[3] Cruz-Jentoft A.J., Landi F., Schneider S.M., Zúñiga C., Arai H., Boirie Y., Chen L.-K., Fielding R.A., Martin F.C., Michel J.-P. (2014). Prevalence of and interventions for sarcopenia in ageing adults: A systematic review. Report of the International Sarcopenia Initiative (EWGSOP and IWGS). Age Ageing.

[4] Lobstein T., Jackson-Leach R., Powis J., Brinsden H., Gray M. (2023). World Obesity Atlas 2023.

[5] Gao Q., Mei F., Shang Y., Hu K., Chen F., Zhao L., Ma B. (2021). Global prevalence of sarcopenic obesity in older adults: A systematic review and meta-analysis. Clin. Nutr..

[6] Luo Y., Shu L., Wang Y., Zhao X., Han M., Liu Y., Xu Y., Han B. (2025). Sarcopenic obesity and risk of cardio-cerebrovascular disease and mortality: A systematic review and meta-analysis. Int. J. Obes..

[7] Gandham A., Mesinovic J., Jansons P., Zengin A., Bonham M.P., Ebeling P.R., Scott D. (2021). Falls, fractures, and areal bone mineral density in older adults with sarcopenic obesity: A systematic review and meta-analysis. Obes. Rev..

[8] Donini L.M., Busetto L., Bischoff S.C., Cederholm T., Ballesteros-Pomar M.D., Batsis J.A., Bauer J.M., Boirie Y., Cruz-Jentoft A.J., Dicker D. (2022). Definition and diagnostic criteria for sarcopenic obesity: ESPEN and EASO consensus statement. Clin. Nutr..

[9] Guimarães N.S., Reis M.G., Tameirão D.R., Cezar N.O.d.C., Leopoldino A.A.O., Magno L.A.V. (2024). Factors associated with sarcopenic obesity in Brazilian adults and older people: Systematic review and meta-analysis of observational studies. Geriatr. Gerontol. Int..

[10] Daskalopoulou C., Wu Y.-T., Pan W., Vázquez I.G., Prince M., Prina M., Tyrovolas S. (2020). Factors related with sarcopenia and sarcopenic obesity among low- and middle-income settings: The 10/66 DRG study. Sci. Rep..

[11] Prokopidis K., Witard O.C. (2022). Understanding the role of smoking and chronic excess alcohol consumption on reduced caloric intake and the development of sarcopenia. Nutr. Res. Rev..

[12] Eglseer D., Traxler M., Schoufour J.D., Weijs P.J.M., Voortman T., Boirie Y., Cruz-Jentoft A.J., Reiter L., Bauer S., for the SO-NUTS Consortium (2023). Nutritional and exercise interventions in individuals with sarcopenic obesity around retirement age: A systematic review and meta-analysis. Nutr. Rev..

[13] Kivimäki M., Batty G.D., Pentti J., Shipley M.J., Sipilä P., Nyberg S.T., Suominen S.B., Oksanen T., Stenholm S., Virtanen M. (2020). Association between socioeconomic status and the development of mental and physical health conditions in adulthood: A multi-cohort study. Lancet Public Health.

[14] Alao R., Nur H., Fivian E., Shankar B., Kadiyala S., Harris-Fry H. (2021). Economic inequality in malnutrition: A global systematic review and meta-analysis. BMJ Glob. Health.

[15] Bann D., Villadsen A., Maddock J., Hughes A., Ploubidis G.B., Silverwood R., Patalay P. (2021). Changes in the behavioural determinants of health during the COVID-19 pandemic: Gender, socioeconomic and ethnic inequalities in five British cohort studies. J. Epidemiol. Community Health.

[16] Prado C.M., Batsis J.A., Donini L.M., Gonzalez M.C., Siervo M. (2024). Sarcopenic obesity in older adults: A clinical overview. Nat. Rev. Endocrinol..

[17] Swan L., Warters A., O’Sullivan M. (2022). Socioeconomic Disadvantage is Associated with Probable Sarcopenia in Community-Dwelling Older Adults: Findings from the English Longitudinal Study of Ageing. J. Frailty Aging.

[18] Anekwe C.V., Jarrell A.R., Townsend M.J., Gaudier G.I., Hiserodt J.M., Stanford F.C. (2020). Socioeconomics of Obesity. Curr. Obes. Rep..

[19] Gandham A., Zengin A., Bonham M.P., Brennan-Olsen S.L., Aitken D., Winzenberg T.M., Ebeling P.R., Jones G., Scott D. (2021). Associations between socioeconomic status and obesity, sarcopenia, and sarcopenic obesity in community-dwelling older adults: The Tasmanian Older Adult Cohort Study. Exp. Gerontol..

[20] Jang W., Kim H. (2023). Association of socioeconomic factors and dietary intake with sarcopenic obesity in the Korean older population. Asia Pac. J. Clin. Nutr..

[21] Kim Y., Han B.-G., the KoGES group (2017). Cohort Profile: The Korean Genome and Epidemiology Study (KoGES) Consortium. Int. J. Epidemiol..

[22] Studenski S.A., Peters K.W., Alley D.E., Cawthon P.M., McLean R.R., Harris T.B., Ferrucci L., Guralnik J.M., Fragala M.S., Kenny A.M. (2014). The FNIH sarcopenia project: Rationale, study description, conference recommendations, and final estimates. J. Gerontol. A Biol. Sci. Med. Sci..

[23] Byeon H.J., Kim Y.J., Yoon J.S., Ko J. (2023). Sarcopenia as a potential risk factor for senile blepharoptosis: Nationwide Surveys (KNHANES 2008–2011). Sci. Rep..

[24] Tan L.F., Chan Y.H., Denishkrshna A., Merchant R.A. (2024). Association between different skeletal muscle mass indices, physical function, and inflammation in obese pre-frail older adults. Arch. Gerontol. Geriatr..

[25] World Health Organization (2000). The Asia-Pacific Perspetive: Redefining Obesity and Its Treatment.

[26] Boshnjaku A., Bahtiri A., Feka K., Krasniqi E., Tschan H., Wessner B. (2022). Impact of Using Population-Specific Cut-Points, Self-Reported Health, and Socio-Economic Parameters to Predict Sarcopenia: A Cross-Sectional Study in Community-Dwelling Kosovans Aged 60 Years and Older. J. Clin. Med..

[27] Estrela M., Semedo G., Roque F., Ferreira P.L., Herdeiro M.T. (2023). Sociodemographic determinants of digital health literacy: A systematic review and meta-analysis. Int. J. Med. Inf..

[28] Zhao J., Huang J., Nie F. (2022). The Income Elasticities of Food, Calories, and Nutrients in China: A Meta-Analysis. Nutrients.

[29] Leung C.Y., Huang H.-L., Abe S.K., Saito E., Islam R., Rahman S., Ikeda A., Sawada N., Tamakoshi A., Gao Y.-T. (2022). Association of Marital Status with Total and Cause-Specific Mortality in Asia. JAMA Netw. Open.

[30] Gao Q., Hu K., Yan C., Zhao B., Mei F., Chen F., Zhao L., Shang Y., Ma Y., Ma B. (2021). Associated Factors of Sarcopenia in Community-Dwelling Older Adults: A Systematic Review and Meta-Analysis. Nutrients.

[31] Islam S., Jaffee S.R. (2024). Social mobility and mental health: A systematic review and meta-analysis. Soc. Sci. Med..

[32] Xu Q., Bu F., Song Z., Li K., Fang C., Luo Y., Zhang L., Pei Y. (2025). Association of serum 25-hydroxyvitamin D with sarcopenic obesity risk: A longitudinal observational study from the UK Biobank. Obesity.

[33] Xu Q., Zhao Q.-G., Ma X.-L., Yan S.-S., Han B.-X., Song Z.-T., Bu F., Li K., Zhang L., Pei Y.-F. (2024). Exome-Wide Sequencing Study Identified Genetic Variants Associated with Sarcopenic Obesity. J. Gerontol. A Biol. Sci. Med. Sci..

[34] Chen T.-P., Kao H.-H., Ogawa W., Arai H., Tahapary D.L., Assantachai P., Tham K.-W., Chan D.-C., Yuen M.M.-A., Appannah G. (2025). The Asia-Oceania consensus: Definitions and diagnostic criteria for sarcopenic obesity. Obes. Res. Clin. Pract..

[35] Kirk B., Cawthon P.M., Arai H., Ávila-Funes J.A., Barazzoni R., Bhasin S., Binder E.F., Bruyere O., Cederholm T., Chen L.-K. (2024). The Conceptual Definition of Sarcopenia: Delphi Consensus from the Global Leadership Initiative in Sarcopenia (GLIS). Age Ageing.

[36] Chun M., Lee S.R. (2025). Association between higher eating frequency and lower odds of low muscle mass in Koreans. Front. Med..

[37] Chen L., Meng L., Peng L., Lee W., Zhang S., Nishita Y., Otsuka R., Yamada M., Pan W., Kamaruzzaman S.B. (2025). Mapping Normative Muscle Health Metrics Across the Aging Continuum: A Multinational Study Pooling Data from Eight Cohorts in Japan, Malaysia and Taiwan. J. Cachexia Sarcopenia Muscle.

