# Supplementary material for: Association Between Socioeconomic Status and Incident Sarcopenic Obesity: A 17-Year Prospective Cohort Study
Source: J Clin Med. 2026 May 15;15(10):3816. doi: 10.3390/jcm15103816 (PMC13207048; doi:10.3390/jcm15103816)
Supplement: Supplementary file 1 [file jcm-15-03816-s001.zip › jcm-4240376-supplementary.pdf]

Supplementary Table S1. Baseline characteristics according to educational attainment

Characteristics	Elementary school (n=2,515)	Middle-High school (n=4,344)	College or higher (n=1,101)	p-value
Age (y)	58.4 ± 7.8	49.0 ± 7.5	47.5 ± 7.3	<0.001
Sex				<0.001
Male	758 (30.1)	2237 (51.5)	836 (75.9)	
Female	1757 (69.9)	2107 (48.5)	265 (24.1)	
Height (cm)	156.3 ± 7.5	162.0 ± 7.9	166.5 ± 7.6	<0.001
Weight (kg)	58.5 ± 9.2	63.5 ± 9.8	67.5 ± 10.6	<0.001
BMI (kg/m²)	23.9 ± 3.0	24.1 ± 2.8	24.5 ± 2.8	<0.001
Waist circumference (cm)	82.9 ± 8.6	81.0 ± 8.3	81.7 ± 8.4	<0.001
Waist/Hip ratio	0.9 ± 0.0	0.9 ± 0.0	0.9 ± 0.0	<0.001
Body fat mass (kg)	16.3 ± 5.4	16.1 ± 5.0	16.0 ± 4.8	0.058
Body fat mass (%)	27.5 ± 7.0	25.3 ± 6.8	23.6 ± 6.0	<0.001
Skeletal muscle mass (kg)	39.8 ± 6.6	44.8 ± 8.0	48.7 ± 8.3	<0.001
SMM/BMI	1.7 ± 0.3	1.9 ± 0.3	2.0 ± 0.3	<0.001
Lean body mass (kg)	42.2 ± 6.9	47.4 ± 8.4	51.5 ± 8.6	<0.001
Systolic BP (mmHg)	126.6 ± 19.2	118.0 ± 16.8	116.7 ± 16.5	<0.001
Diastolic BP (mmHg)	81.9 ± 11.3	78.8 ± 11.2	78.7 ± 11.3	<0.001
Glucose (mg/dL)	85.5 ± 19.0	86.5 ± 19.8	89.5 ± 24.4	<0.001
HbA1c (%)	5.9 ± 0.9	5.7 ± 0.9	5.7 ± 0.9	<0.001
Total cholesterol (mg/dL)	188.3 ± 35.4	188.4 ± 34.6	198.5 ± 35.6	<0.001
HDL cholesterol (mg/dL)	44.8 ± 10.2	45.0 ± 10.0	44.5 ± 9.9	0.641
LDL cholesterol (mg/dL)	111.7 ± 32.4	112.6 ± 31.9	121.4 ± 33.0	<0.001
Triglyceride (mg/dL)	161.0 ± 94.1	155.9 ± 103.9	164.1 ± 105.0	0.997
Hypertension	996 (39.7)	1078 (24.9)	286 (26.0)	<0.001
Diabetes mellitus	354 (14.2)	406 (9.4)	125 (11.4)	<0.001
Dyslipidemia	24 (1.0)	110 (2.5)	52 (4.7)	<0.001
Drinking status				<0.001
Non-drinker	1463 (58.7)	1765 (40.9)	356 (32.3)	
Ex-drinker	148 (5.9)	283 (6.6)	60 (5.5)	
Current drinker	882 (35.4)	2268 (52.6)	685 (62.2)	
Smoking status				<0.001
Non-smoker	1764 (71.1)	2377 (55.3)	427 (38.9)	
Ex-smoker	239 (9.6)	701 (16.3)	289 (26.3)	
Current smoker	478 (19.3)	1224 (28.5)	383 (34.9)	
Exercise				<0.001
Yes	789 (32.6)	1632 (38.5)	483 (44.4)	
No	1635 (67.5)	2606 (61.5)	606 (55.7)	
Caloric intake (kcal)	1855.8 ± 761.0	2000.1 ± 707.4	2073.6 ± 613.8	<0.001
Protein intake (g)	59.4 ± 31.2	69.4 ± 30.5	74.2 ± 27.2	<0.001
Fat intake (g)	25.9 ± 20.7	35.4 ± 21.8	39.3 ± 19.9	<0.001
Carbohydrate intake (g)	341.3 ± 132.3	346.3 ± 116.0	351.1 ± 99.8	0.017

Household income (million KRW)[a]				<0.001
<1.0	1627 (66.1)	946 (22.1)	100 (9.1)	
1.0 – 3.9	804 (32.6)	2974 (69.3)	780 (71.3)	
≥4.0	32 (1.3)	369 (8.6)	214 (19.6)	
Marital status				<0.001
Unmarried[b]	427 (17.2)	233 (5.4)	49 (4.5)	
Married	2059 (82.8)	4099 (94.6)	1052 (95.6)	

Abbreviations: BMI, body mass index; SMM/BMI, BMI-adjusted skeletal muscle mass index; BP, blood pressure; HbA1c, glycated hemoglobin; KRW, korean won

Differences in sex distribution across educational levels may reflect the recruitment characteristics of the Ansan and Anseong cohorts during 2001–2003 rather than the underlying population structure.

Data are presented as mean ± standard deviation for continuous variables and number (percentage) for categorical variables.

[a]For example, 4.0 = 4,000,000 KRW

[b]Unmarried status included individuals who were single, divorced, separated, or widowed.

p-values were calculated using the ANOVA for continuous variables and Chi-square test for categorical variables.

Supplementary Table S2. Baseline characteristics according to household income categories (million KRW)[a]

Characteristics	<1.0 (n=2,702)	1.0 – 3.9 (n=4,570)	≥4.0 (n=617)	p-value
Age (y)	57.7 ± 8.3	49.0 ± 7.6	46.7 ± 6.0	<0.001
Sex				<0.001
Male	1054 (39.0)	2387 (52.2)	373 (60.5)	
Female	1648 (61.0)	2183 (47.8)	244 (39.6)	
Height (cm)	157.8 ± 8.1	162.1 ± 8.2	164.7 ± 8.3	<0.001
Weight (kg)	59.2 ± 9.4	63.8 ± 10.0	67.5 ± 11.0	<0.001
BMI (kg/m²)	23.7 ± 3.0	24.2 ± 2.8	24.8 ± 2.8	<0.001
Waist circumference (cm)	82.7 ± 8.6	81.1 ± 8.4	82.0 ± 8.3	<0.001
Waist/Hip ratio	0.9 ± 0.0	0.9 ± 0.0	0.9 ± 0.0	<0.001
Body fat mass (kg)	15.8 ± 5.4	16.2 ± 5.0	17.2 ± 4.9	<0.001
Body fat mass (%)	26.5 ± 7.2	25.4 ± 6.8	25.5 ± 6.1	<0.001
Skeletal muscle mass (kg)	41.0 ± 7.1	44.9 ± 8.2	47.5 ± 8.7	<0.001
SMM/BMI	1.7 ± 0.3	1.9 ± 0.3	1.9 ± 0.3	<0.001
Lean body mass (kg)	43.4 ± 7.5	47.6 ± 8.6	50.3 ± 9.1	<0.001
Systolic BP (mmHg)	126.0 ± 18.5	118.0 ± 17.4	115.6 ± 15.6	<0.001
Diastolic BP (mmHg)	81.7 ± 11.0	78.8 ± 11.3	78.7 ± 11.4	<0.001
Glucose (mg/dL)	85.3 ± 18.7	86.9 ± 20.9	90.7 ± 24.1	<0.001
HbA1c (%)	5.8 ± 0.9	5.7 ± 0.9	5.7 ± 0.9	<0.001
Total cholesterol (mg/dL)	186.8 ± 35.5	190.6 ± 34.5	198.2 ± 36.8	<0.001
HDL cholesterol (mg/dL)	45.1 ± 10.3	44.8 ± 9.9	44.6 ± 9.9	0.143
LDL cholesterol (mg/dL)	109.9 ± 32.4	114.7 ± 31.7	121.9 ± 34.9	<0.001
Triglyceride (mg/dL)	160.9 ± 97.1	156.5 ± 101.0	164.0 ± 115.4	0.590
Hypertension	1041 (38.6)	1155 (25.3)	145 (23.5)	<0.001
Diabetes mellitus	375 (14.0)	434 (9.5)	66 (10.7)	<0.001
Dyslipidemia	36 (1.3)	124 (2.7)	25 (4.1)	<0.001
Drinking status				<0.001

Non-drinker	1455 (54.4)	1874 (41.2)	215 (34.9)	
Ex-drinker	176 (6.6)	273 (6.0)	34 (5.5)	
Current drinker	1044 (39.0)	2404 (52.8)	368 (59.6)	
Smoking status				<0.001
Non-smoker	1705 (63.8)	2517 (55.5)	298 (48.5)	
Ex-smoker	308 (11.5)	781 (17.2)	138 (22.4)	
Current smoker	658 (24.6)	1237 (27.3)	179 (29.1)	
Exercise				<0.001
Yes	861 (33.1)	1766 (39.4)	257 (41.9)	
No	1739 (66.9)	2713 (60.6)	356 (58.1)	
Caloric intake (kcal)	1900.2 ± 798.9	1983.5 ± 647.5	2075.7 ± 694.9	<0.001
Protein intake (g)	61.1 ± 33.0	68.9 ± 27.2	76.6 ± 36.7	<0.001
Fat intake (g)	27.4 ± 22.1	35.1 ± 19.8	40.6 ± 26.0	<0.001
Carbohydrate intake (g)	347.3 ± 136.4	343.3 ± 108.5	346.4 ± 95.6	0.392
Education				<0.001
Elementary school	1627 (60.9)	804 (17.6)	32 (5.2)	
Middle-High school	946 (35.4)	2974 (65.3)	369 (60.0)	
College or higher	100 (3.7)	780 (17.1)	214 (34.8)	
Marital status				<0.001
Unmarried[b]	428 (16.0)	260 (5.7)	10 (1.6)	
Married	2250 (84.0)	4296 (94.3)	606 (98.4)	

Abbreviations: KRW, korean won; BMI, body mass index; SMM/BMI, BMI-adjusted skeletal muscle mass index; BP, blood pressure; HbA1c, glycated hemoglobin

Data are presented as mean ± standard deviation for continuous variables and number (percentage) for categorical variables.

[a]For example, 4.0 = 4,000,000 KRW

[b]Unmarried status included individuals who were single, divorced, separated, or widowed.

p-values were calculated using the ANOVA for continuous variables and Chi-square test for categorical variables.

Supplementary Table S3. Baseline characteristics according to marital status

Characteristics	Unmarried[a] (n=716)	Married (n=7,247)	p-value
Age (y)	57.2 ± 9.0	51.3 ± 8.6	<0.001
Sex			<0.001
Male	154 (21.5)	3677 (50.7)	
Female	562 (78.5)	3570 (49.3)	
Height (cm)	156.0 ± 7.7	161.3 ± 8.4	<0.001
Weight (kg)	58.5 ± 9.3	62.9 ± 10.2	<0.001
BMI (kg/m^2)	24.0 ± 2.9	24.1 ± 2.9	0.388
Waist circumference (cm)	82.3 ± 9.1	81.7 ± 8.4	0.047
Waist/Hip ratio	0.9 ± 0.0	0.9 ± 0.0	<0.001
Body fat mass (kg)	16.9 ± 5.3	16.1 ± 5.1	<0.001
Body fat mass (%)	28.7 ± 6.7	25.5 ± 6.8	<0.001
Skeletal muscle mass (kg)	39.3 ± 6.9	44.2 ± 8.2	<0.001
SMM/BMI	1.7 ± 0.3	1.8 ± 0.3	<0.001

Lean body mass (kg)	41.7 ± 7.2	46.8 ± 8.5	<0.001
Systolic BP (mmHg)	124.4 ± 19.5	120.2 ± 17.9	<0.001
Diastolic BP (mmHg)	80.7 ± 11.9	79.7 ± 11.3	0.025
Glucose (mg/dL)	86.7 ± 19.0	86.7 ± 20.6	0.923
HbA1c (%)	5.9 ± 1.1	5.7 ± 0.9	<0.001
Total cholesterol (mg/dL)	193.6 ± 35.7	189.4 ± 35.1	0.003
HDL cholesterol (mg/dL)	45.5 ± 10.3	44.8 ± 10.0	0.093
LDL cholesterol (mg/dL)	115.7 ± 32.7	113.4 ± 32.3	0.066
Triglyceride (mg/dL)	163.1 ± 94.3	157.9 ± 101.4	0.191
Hypertension	265 (37.1)	2099 (29.0)	<0.001
Diabetes mellitus	112 (15.7)	774 (10.7)	<0.001
Dyslipidemia	11 (1.5)	175 (2.4)	0.175
Drinking status			<0.001
Non-drinker	446 (62.9)	3154 (43.8)	
Ex-drinker	29 (4.1)	458 (6.4)	
Current drinker	234 (33.0)	3594 (49.9)	
Smoking status			<0.001
Non-smoker	523 (73.9)	4047 (56.4)	
Ex-smoker	46 (6.5)	1185 (16.5)	
Current smoker	139 (19.6)	1945 (27.1)	
Exercise			<0.001
Yes	235 (33.7)	2666 (37.8)	
No	463 (66.3)	4395 (62.2)	
Caloric intake (kcal)	1846.4 ± 708.6	1975.3 ± 717.1	<0.001
Protein intake (g)	59.4 ± 27.0	67.6 ± 31.0	<0.001
Fat intake (g)	27.2 ± 20.2	33.5 ± 21.9	<0.001
Carbohydrate intake (g)	336.0 ± 123.8	346.0 ± 118.8	0.036
Education			<0.001
Elementary school	427 (60.2)	2059 (28.6)	
Middle-High school	233 (32.9)	4099 (56.9)	
College or higher	49 (6.9)	1052 (14.6)	
Household income (million KRW)[b]			<0.001
<1.0	428 (61.3)	2250 (31.5)	
1.0 – 3.9	260 (37.3)	4296 (60.1)	
≥4.0	10 (1.4)	606 (8.5)	

Abbreviations: BMI, body mass index; SMM/BMI, BMI-adjusted skeletal muscle mass index; BP, blood pressure; HbA1c, glycated hemoglobin; KRW, korean won

Data are presented as mean ± standard deviation for continuous variables and number (percentage) for categorical variables.

[a]Unmarried status included individuals who were single, divorced, separated, or widowed.

[b]For example, 4.0 = 4,000,000 KRW

p-values were calculated using the ANOVA for continuous variables and Chi-square test for categorical variables.

Supplementary Table S4. Multivariable-adjusted hazard ratios for new onset of sarcopenic obesity by socioeconomic status in men[a]

	Event	Model 1		Model 2		Model 3	
		HR (95% CI)	p-value	HR (95% CI)	p-value	HR (95% CI)	p-value
Education							
Elementary school	98 (12.9)	1.04 (0.77, 1.41)	0.806	1.03 (0.76, 1.41)	0.835	1.03 (0.75, 1.41)	0.846
Middle-High school	312 (14.0)	1.14 (0.91, 1.44)	0.253	1.14 (0.90, 1.44)	0.276	1.14 (0.90, 1.44)	0.292
College or higher	97 (11.6)	reference		reference		reference	
Household income (million KRW)[b]							
<1.0	140 (13.3)	1.31 (0.90, 1.92)	0.159	1.29 (0.88, 1.89)	0.195	1.30 (0.88, 1.91)	0.183
1.0 – 3.9	327 (13.7)	1.38 (0.99, 1.94)	0.060	1.35 (0.96, 1.90)	0.080	1.37 (0.98, 1.93)	0.069
≥4.0	38 (10.2)	reference		reference		reference	
Marital status							
Unmarried[c]	25 (16.2)	1.34 (0.90, 2.00)	0.153	1.42 (0.95, 2.13)	0.088	1.47 (0.98, 2.20)	0.063
Married	484 (13.2)	reference		reference		reference	

Abbreviation: HR, hazard ratio; CI, confidence interval; KRW, korean won

Data are presented as number (percentage) and hazard ratio with 95% confidence interval.

[a]Event indicates the number of cases.

[b]For example, 4.0 = 4,000,000 KRW

[c]Unmarried status included individuals who were single, divorced, separated, or widowed.

p-values were derived from the Wald test in Cox proportional hazards models.

Model 1: adjusted for age.

Model 2: adjusted for age, smoking, drinking, exercise and daily protein intake.

Model 3: adjusted for age, smoking, drinking, exercise, daily protein intake, hypertension, dyslipidemia and diabetes mellitus.

Supplementary Table S5. Multivariable-adjusted hazard ratios for new onset of sarcopenic obesity by socioeconomic status in women[a]

	Event	Model 1		Model 2		Model 3	
		HR (95% CI)	p-value	HR (95% CI)	p-value	HR (95% CI)	p-value
Education							
Elementary school	436 (24.8)	1.75 (1.22, 2.50)	0.002	1.69 (1.16, 2.45)	0.006	1.63 (1.12, 2.37)	0.010
Middle-High school	303 (14.4)	1.09 (0.77, 1.54)	0.646	1.10 (0.77, 1.58)	0.595	1.10 (0.76, 1.57)	0.619
College or higher	35 (13.2)	reference		reference		reference	
Household income (million KRW)[b]							
<1.0	382 (23.2)	1.29 (0.92, 1.83)	0.141	1.24 (0.87, 1.76)	0.234	1.19 (0.84, 1.70)	0.330
1.0 – 3.9	345 (15.8)	0.98 (0.70, 1.36)	0.887	0.92 (0.65, 1.29)	0.621	0.91 (0.65, 1.27)	0.572

≥4.0	39 (16.0)	reference		reference		reference	
Marital status							
Unmarried[c]	130 (23.1)	1.24 (1.01, 1.50)	0.036	1.21 (0.99, 1.49)	0.061	1.22 (1.00, 1.50)	0.056
Married	646 (18.1)	reference		reference		reference	

Abbreviation: HR, hazard ratio; CI, confidence interval; KRW, korean won

Data are presented as number (percentage) and hazard ratio with 95% confidence interval.

[a]Event indicates the number of cases.

[b]For example, 4.0 = 4,000,000 KRW

[c]Unmarried status included individuals who were single, divorced, separated, or widowed.

p-values were derived from the Wald test in Cox proportional hazards models.

Model 1: adjusted for age.

Model 2: adjusted for age, smoking, drinking, exercise and daily protein intake.

Model 3: adjusted for age, smoking, drinking, exercise, daily protein intake, hypertension, dyslipidemia and diabetes mellitus.

Supplementary Table S6. Multivariable-adjusted hazard ratios for new onset of sarcopenic obesity by socioeconomic status by age group[a]

40-49 years	Event	Model 1		Model 2		Model 3	
		HR (95% CI)	p-value	HR (95% CI)	p-value	HR (95% CI)	p-value
Education							
Elementary school	88 (20.1)	1.84 (1.35, 2.50)	<0.001	1.87 (1.36, 2.57)	<0.001	1.81 (1.31, 2.49)	<0.001
Middle-High school	347 (12.8)	1.15 (0.90, 1.46)	0.264	1.15 (0.90, 1.47)	0.274	1.15 (0.89, 1.47)	0.280
College or higher	85 (10.8)	reference		reference		reference	
Household income (million KRW)[b]							
<1.0	93 (16.2)	1.64 (1.16, 2.31)	0.005	1.59 (1.12, 2.27)	0.010	1.59 (1.11, 2.26)	0.011
1.0 – 3.9	371 (13.0)	1.28 (0.96, 1.72)	0.096	1.22 (0.91, 1.65)	0.191	1.24 (0.92, 1.67)	0.162
≥4.0	51 (10.7)	reference		reference		reference	
Marital status							
Unmarried[c]	29 (15.4)	1.29 (0.89, 1.88)	0.178	1.33 (0.91, 1.95)	0.144	1.36 (0.93, 2.00)	0.113
Married	488 (13.0)	reference		reference		reference	
**50-59 years**	**Event**	**Model 1**		**Model 2**		**Model 3**	
		HR (95% CI)	p-value	HR (95% CI)	p-value	HR (95% CI)	p-value
Education							
Elementary school	177 (22.7)	1.19 (0.81, 1.74)	0.372	1.13 (0.76, 1.69)	0.540	1.10 (0.74, 1.64)	0.645
Middle-High school	181 (16.8)	0.94 (0.65, 1.36)	0.751	0.95 (0.65, 1.39)	0.794	0.93 (0.64, 1.37)	0.730
College or higher	34 (16.3)	reference		reference		reference	
Household income (million KRW)[b]							

<1.0	183 (22.8)	1.44 (0.87, 2.37)	0.156	1.39 (0.84, 2.30)	0.200	1.34 (0.81, 2.22)	0.261
1.0 – 3.9	188 (16.7)	1.06 (0.64, 1.74)	0.825	1.06 (0.64, 1.74)	0.821	1.03 (0.63, 1.70)	0.897
≥4.0	17 (15.7)	reference		reference		reference	
Marital status							
Unmarried[c]	42 (26.3)	1.36 (0.98, 1.88)	0.065	1.31 (0.94, 1.83)	0.114	1.31 (0.94, 1.84)	0.110
Married	353 (18.5)	reference		reference		reference	

≥60 years	Event	**Model 1**		**Model 2**		**Model 3**	
		HR (95% CI)	p-value	HR (95% CI)	p-value	HR (95% CI)	p-value
Education							
Elementary school	269 (20.7)	1.37 (0.77, 2.41)	0.281	1.38 (0.77, 2.44)	0.276	1.40 (0.79, 2.50)	0.248
Middle-High school	87 (15.6)	1.18 (0.66, 2.11)	0.585	1.23 (0.68, 2.21)	0.500	1.26 (0.70, 2.26)	0.450
College or higher	13 (12.4)	reference		reference		reference	
Household income (million KRW)[b]							
<1.0	246 (18.6)	0.63 (0.32, 1.22)	0.169	0.64 (0.33, 1.25)	0.189	0.61 (0.31, 1.19)	0.146
1.0 – 3.9	113 (19.1)	0.67 (0.34, 1.32)	0.250	0.65 (0.33, 1.29)	0.221	0.63 (0.32, 1.25)	0.190
≥4.0	9 (27.3)	reference		reference		reference	
Marital status							
Unmarried[c]	84 (22.8)	1.16 (0.90, 1.50)	0.241	1.19 (0.91, 1.54)	0.205	1.18 (0.91, 1.54)	0.214
Married	289 (18.1)	reference		reference		reference	

Abbreviation: HR, hazard ratio; CI, confidence interval; KRW, korean won

Data are presented as number (percentage) and hazard ratio with 95% confidence interval.

Age groups were categorized as 40–49, 50–59, and ≥60 years.

[a]Event indicates the number of cases.

[b]For example, 4.0 = 4,000,000 KRW

[c]Unmarried status included individuals who were single, divorced, separated, or widowed.

p-values were derived from the Wald test in Cox proportional hazards models.

Model 1: adjusted for sex.

Model 2: adjusted for sex, smoking, drinking, exercise and daily protein intake.

Model 3: adjusted for sex, smoking, drinking, exercise, daily protein intake, hypertension, dyslipidemia and diabetes mellitus.
